# Restriction Checkpoint Controls Bradyzoite Development in Toxoplasma gondii

**DOI:** 10.1128/spectrum.00702-22

**Published:** 2022-06-02

**Authors:** Anatoli V. Naumov, Chengqi Wang, Dale Chaput, Li-Min Ting, Carmelo A. Alvarez, Thomas Keller, Ahmed Ramadan, Michael W. White, Kami Kim, Elena S. Suvorova

**Affiliations:** a Department of Internal Medicine, Division of Infectious Diseases and International Medicine, Morsani College of Medicine, University of South Florida, Tampa, Florida, USA; b Center for Global Health and Infectious Diseases Research and USF Genomics Program, College of Public Health, University of South Florida, Tampa, Florida, USA; c Proteomics Core, College of Arts and Sciences, University of South Florida, Tampa, Florida, USA; d Department of Molecular Medicine, Morsani College of Medicine, University of South Florida, Tampa, Florida, USA; Weill Cornell Medicine

**Keywords:** apicomplexan, *Toxoplasma gondii*, bradyzoite, development, cyclin, cyclin-dependent kinase

## Abstract

Human toxoplasmosis is a life-threatening disease caused by the apicomplexan parasite Toxoplasma gondii. Rapid replication of the tachyzoite is associated with symptomatic disease, while suppressed division of the bradyzoite is responsible for chronic disease. Here, we identified the T. gondii cell cycle mechanism, the G_1_ restriction checkpoint (R-point), that operates the switch between parasite growth and differentiation. Apicomplexans lack conventional R-point regulators, suggesting adaptation of alternative factors. We showed that Cdk-related G_1_ kinase TgCrk2 forms alternative complexes with atypical cyclins (TgCycP1, TgCycP2, and TgCyc5) in the rapidly dividing developmentally incompetent RH and slower dividing developmentally competent ME49 tachyzoites and bradyzoites. Examination of cyclins verified the correlation of cyclin expression with growth dependence and development capacity of RH and ME49 strains. We demonstrated that rapidly dividing RH tachyzoites were dependent on TgCycP1 expression, which interfered with bradyzoite differentiation. Using the conditional knockdown model, we established that TgCycP2 regulated G_1_ duration in the developmentally competent ME49 tachyzoites but not in the developmentally incompetent RH tachyzoites. We tested the functions of TgCycP2 and TgCyc5 in alkaline induced and spontaneous bradyzoite differentiation (rat embryonic brain cells) models. Based on functional and global gene expression analyses, we determined that TgCycP2 also regulated bradyzoite replication, while signal-induced TgCyc5 was critical for efficient tissue cyst maturation. In conclusion, we identified the central machinery of the T. gondii restriction checkpoint comprised of TgCrk2 kinase and three atypical T. gondii cyclins and demonstrated the independent roles of TgCycP1, TgCycP2, and TgCyc5 in parasite growth and development.

**IMPORTANCE** Toxoplasma gondii is a virulent and abundant human pathogen that puts millions of silently infected people at risk of reactivation of the chronic disease. Encysted bradyzoites formed during the chronic stage are resistant to current therapies. Therefore, insights into the mechanism of tissue cyst formation and reactivation are major areas of investigation. The fact that rapidly dividing parasites differentiate poorly strongly suggests that there is a threshold of replication rate that must be crossed to be considered for differentiation. We discovered a cell cycle mechanism that controls the T. gondii growth-rest switch involved in the conversion of dividing tachyzoites into largely quiescent bradyzoites. This switch operates the T. gondii restriction checkpoint using a set of atypical and parasite-specific regulators. Importantly, the novel T. gondii R-point network was not present in the parasite's human and animal hosts, offering a wealth of new and parasite-specific drug targets to explore in the future.

## INTRODUCTION

**FIG 1:**
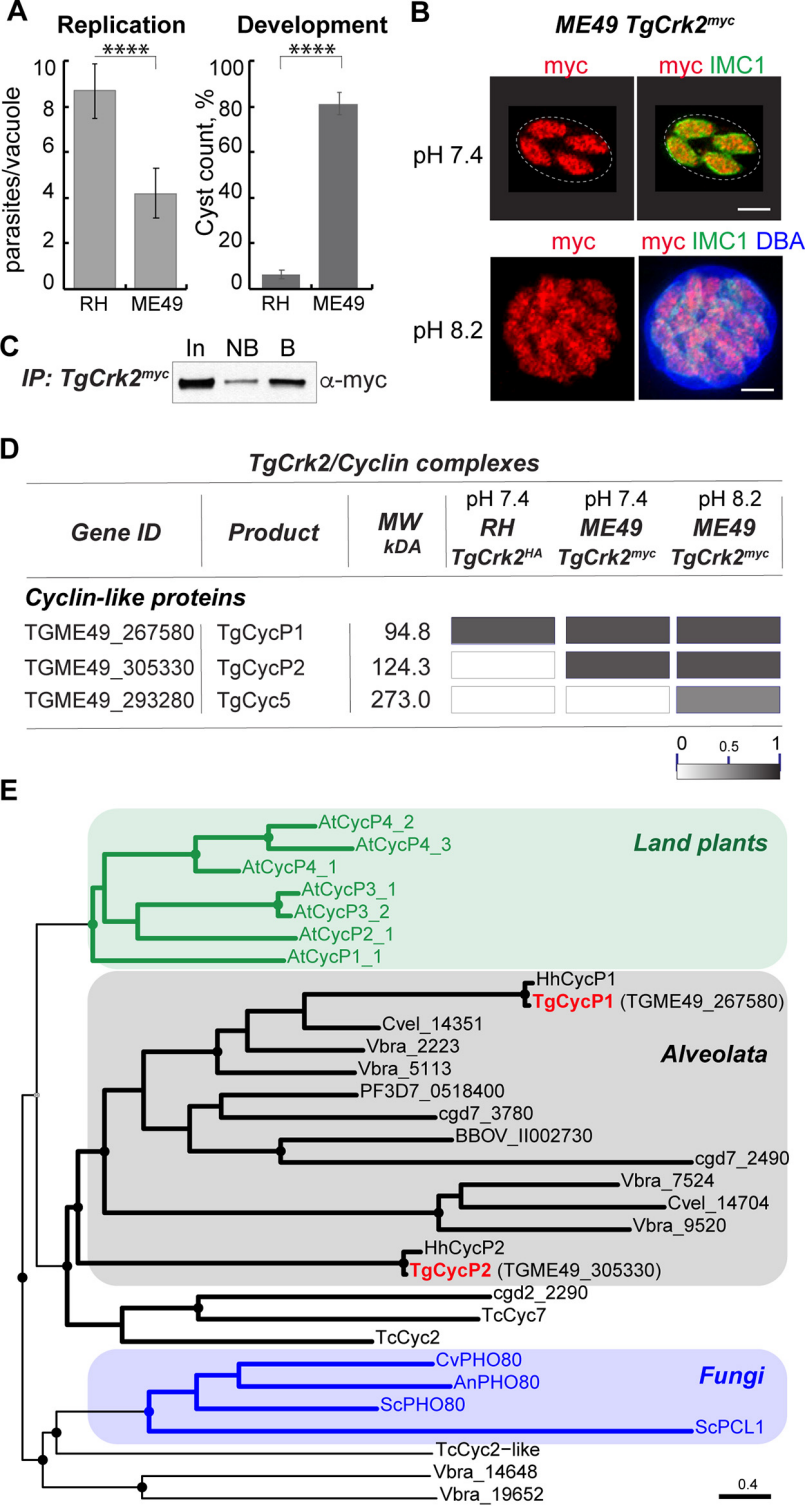
TgCrk2 interacts with three atypical cyclins. (A) Replication and differentiation rates of the parental RHΔ*Ku80TIR1* and ME49Δ*Ku80TIR1* strains. Replication rates are shown on the left graph as the number of the parasites per vacuole after 1-day growth in pH 7.4 medium, 5%CO_2_. The percentage of the DBA-positive cysts after 3 days of growth in pH 8.2 medium, ambient CO_2_ conditions represents the rate of bradyzoite differentiation and is shown on the right graph. Mean values ± SD of three replicates are shown. Unpaired *t* test returned *P* values of 6.4 × 10^−4^ and 8.8 × 10^−6^. (B) Immunofluorescent images of TgCrk2^myc^ expressed in ME49Δ*Ku80TIR1* tachyzoites (pH 7.4 medium, 2 days) or developing bradyzoites (pH 8.2 medium, 3 days). Parasites were labeled with α-myc/α-rabbit IgG Fluor 568, α-IMC1/α-mouse IgG Fluor 488. Developing bradyzoites were identified by costaining with DBA/7-amino-4-methylcoumarin-3-acetic acid methanethiosulfonate (AMCA) Streptavidin 350. Scale bar 5 μm. (C) Immunoblot image of TgCrk2 isolated from ME49Δ*Ku80TIR1* TgCrk2^myc^ tachyzoites grown in pH 7.4 medium at 5% CO_2_. Equal fractions of the soluble proteins pre- (IN: input) and post pulldown (NB: not bound), and immunoprecipitated TgCrk2^myc^ complexes (B: bound) were analyzed. Western blot was probed with α-myc (α-mouse IgG-HRP) to detect TgCrk2^myc^ and confirm pulldown efficiency. (D) Summary of the mass-spectrometry analysis of TgCrk2 complexes purified from RH and ME49 parasites. RHΔ*Ku80* TgCrk2^HA^ and ME49Δ*Ku80TIR1* TgCrk2^myc^ tachyzoites were grown in pH 7.4 medium at 5% CO_2_ for 2 days and ME49Δ*Ku80TIR1* TgCrk2^myc^ bradyzoites were grown in pH 8.2 medium in ambient CO_2_ for 3 days. Table shows detected cyclin proteins. The intensity of the gray bars reflects the probability of cyclin interaction with TgCrk2 kinase. (E) Phylogenetic analysis of apicomplexan P/U type cyclins. Protein sequences of cyclins related to P/U or PHO80 type from representatives of the superphylum alveolate: Toxoplasma gondii (Tg), Hammondia hammondia (Hh), Plasmodium falciparum (PF3D7), Babesia bovis (BBOV), Cryptosporidium parvum (Cgd), Chromera velia (Cvel), Vitrella brassicaformis (Vbra); kinetoplastids: Trypanosome cruzi (Tc); fungi: Saccharomyces cerevisiae (Sc), Candida viswanathii (Cv), Aspergillus niger (An); and land plants: Arabidopsis thaliana (At) were analyzed in R package ‘phangorn’. Branch support was determined in 100 bootstraps and the nodes supported by a higher than 82% value are indicated with a filled circle. T. gondii P-cyclins are shown in red.

Toxoplasma gondii is a tissue cyst forming parasite responsible for a lifelong infection in ~1/3 of the human population. The life cycle of T. gondii consists of periods of active replication and rest, which correlate with the natural progression of the disease. Toxoplasmosis is generally manifested as an acute and chronic infection (1). The rapid division of tachyzoites characterizes the acute phase of the disease. Current drug regimens and a healthy immune system effectively eliminate tachyzoites from an infected host (2). Parasites drastically reduce division during differentiation into transmissive encysted bradyzoites that are resistant to modern therapies (2, 3). In the chronic stage, a host’s weakened immune system allows quiescent bradyzoites to convert into the replicating tachyzoites, which leads to tissue cyst amplification and potential systemic disease in the host (4). It has long been established that T. gondii differentiation is always associated with a reduction in replication rates (5–7). Furthermore, the inverse correlation between replication and differentiation is reflected in the growth characteristics of the three major genetic lineages of T. gondii that differ in virulence and the ability to form tissue cysts (8, 9). Highly virulent type I tachyzoites (e.g., RH, GT1 strains) rapidly divide but cannot efficiently convert into bradyzoites (Fig. 1A and Table S1). In contrast, developmentally competent type II (e.g., ME49, Pru strains) and type III (e.g, VEG, CTG strains) tachyzoites replicate relatively slowly (Fig. 1A and Table S1) (10). Currently, the molecular mechanisms that control the growth-to-rest decision in parasites are not well understood.

Bradyzoite development is a unique process that operates as a rheostat to gradually introduce changes over several rounds of cell division (11–13). Recent studies of toxoplasmosis in mice demonstrated that during bradyzoite development the number of the dividing parasites steadily declines, while the number of parasites in a deep dormant state gradually grows (13). The tachyzoite-to-bradyzoite conversion occurs asynchronously and produces heterogenous tissue cysts comprised of clonal parasites in various physiological states. Single-cell RNA-seq analysis of *in vitro* cysts revealed that the vast majority of the developing bradyzoites are retained in the G_1_ period of the cell cycle (14, 15). Further examination showed that the G_1_ phase is the main source of diversity in bradyzoites (15). Based on their distinctive transcriptome profiles, G_1_ bradyzoites were segregated into a few clusters that likely reflect the physiological diversity of the developing parasites. Previous studies of cell cycle regulators and global transcriptomes verified drastic changes in the bradyzoite cell cycle characterized by an elongated G_1_ period and a longer division cycle (6, 15–19). The tachyzoite and bradyzoite spend vastly different lengths of time in the G_1_ phase (6, 15, 18). The short tachyzoite G_1_ phase (~4 h) provides unobstructed entry into S-phase (division) and is associated with rapid replication that is characteristic of acute disease (16). Developing bradyzoites have a progressively longer G_1_ phase over rounds of cell division (from hours to days/weeks). Mature bradyzoites appear to be retained in the G_1_ phase almost indefinitely and rarely divide (8, 12, 13, 19).

Several surveillance mechanisms regulate eukaryotic cell division. One of such mechanisms is the G_1_ restriction checkpoint (or R-point) that monitors entry into the DNA replication phase and, thereby, warrants cell division. The exclusive function of this checkpoint is to connect signals from the environment to the central biosynthetic pathways of the cell. The restriction checkpoint offers the time necessary to adjust to environmental changes. The conventional R-point is regulated by cyclin-dependent kinases 4/6 and d-type cyclins that are not encoded in apicomplexan genomes (20). Nevertheless, apicomplexan parasites retain a functional restriction checkpoint because quiescent stages, such as sporozoites or mature T. gondii bradyzoites, are suspended in the G_1_ state (6). This suggests the engagement of alternative regulators to control canonical cell cycle functions (11).

Gradual bradyzoite development involves several rounds of cell division and requires unique molecular machinery capable of the stepwise control of cell replication. We recently demonstrated that the tachyzoite G_1_ phase has a definitive stop regulated by atypical Cdk-related kinase TgCrk2 and noncanonical P-type cyclin TgCycP1 (formerly called TgPHO80) (21). Here, we showed that in addition to TgCycP1 (TGME49_267580), kinase TgCrk2 forms complexes with atypical cyclins TgCycP2 (TGME49_305330) and TgCyc5 (TGME49_293280) (21–23). The role of the novel TgCycP2 and TgCyc5 cyclins was examined in the developmentally incompetent RH and competent ME49 strains. Our findings suggested that alternative TgCrk2/Cyclin complexes control the gradual conversion of tachyzoites into bradyzoites. Specifically, we demonstrated that T. gondii cyclins TgCycP1, TgCycP2, and TgCyc5 were the restriction checkpoint regulators and have distinct and critical roles in parasite replication and development.

## RESULTS

### *Toxoplasma* G_1_ kinase TgCrk2 interacted with multiple cyclins.

We recently established that the *Toxoplasma* G_1_ kinase TgCrk2 forms a dominant complex with cyclin TgCycP1 in rapidly dividing RH tachyzoites (21). To find out if TgCrk2 forms a similar complex in developmentally competent type II parasites, we constructed an ME49 strain expressing endogenous TgCrk2 kinase fused with triple Myc-epitope (Fig. 1B). Similar to observed in RH tachyzoites, TgCrk2^myc^ kinase was abundantly and constitutively expressed in ME49 tachyzoites and developing bradyzoites (Fig. 1B). To identify major cyclin partners of this kinase, we examined TgCrk2^myc^ complexes isolated from ME49 tachyzoites and compared them to the complexes formed by TgCrk2^HA^ in RH tachyzoites (Fig. 1C) (21). Proteomic analysis revealed multiple interactions and the most prominent TgCrk2 partners were determined based on the posterior probability of complex formation (Table S2). Corroborating our published results, TgCycP1 was the dominant partner of TgCrk2 in rapidly dividing RH tachyzoites (Fig. 1D). Analysis of developmentally competent ME49 tachyzoites revealed that TgCrk2 kinase formed an equivalent complex with another P-cyclin TgCycP2 (TGME49_305330) (22). To find out whether the TgCrk2 cyclin partners change during bradyzoite development, TgCrk2^myc^ complexes were isolated from the ME49 strain that was grown for 3 days under alkaline conditions. Because these conditions stimulate early bradyzoite development, the analyzed population was comprised of parasites at different stages of the development, including tachyzoites and prebradyzoites (24). We found that in addition to interaction with TgCycP1 and TgCycP2, TgCrk2^myc^ kinase formed a specific complex with the atypical cyclin TgCyc5 (TGME49_293280) (Fig. 1D) (22). While interaction with TgCycP1 and TgCycP2 may have been attributed to the presence of tachyzoites and early-stage bradyzoites, the TgCrk2/TgCyc5 complex was formed only under bradyzoite-induction conditions.

P/U family cyclins have been found in a limited number of eukaryotes, including fungi, plants, and protozoa (23, 25–28). P-cyclins are typically encoded in multiple copies and are known to regulate processes, such as cell cycle progression and environmental sensing. In contrast, apicomplexan parasites retained a small repertoire of P-cyclins: a single gene in haemosporidian and piroplasmids, two genes in Coccidian genera, and multiple P-cyclin genes in cryptosporidia (Table S3) (22, 23). To define the phylogenetic relationships of apicomplexan P-cyclins, we analyzed representative factors in the Apicomplexa phylum, kinetoplastids, and ancestral alveolate Chromerids, and compared them to fungi and land plant P-cyclins (Fig. 1E and Table S3). The results showed that the apicomplexan P-cyclins group with P-cyclins in alveolates differ significantly from plant and fungi P-cyclins. T. gondii genome encodes two P-cyclins, TGME49_267580 (TgCycP1) and TGME49_305330 (TgCycP2). While TgCycP1 has orthologs in all tested apicomplexans and alveolates, factors related to TgCycP2 were only conserved in ancestral Chromerids. Altogether, our phylogenetic and proteomic studies showed that the *Toxoplasma* restriction checkpoint kinase TgCrk2 interacts with multiple atypical cyclins. The dominance of these complexes depends on the parasite’s developmental stage, which suggests differing roles for cyclins TgCycP1, TgCycP2, and TgCyc5 in *Toxoplasma* replication and development.

### TgCycP1 negatively affected bradyzoite differentiation.

**FIG 2:**
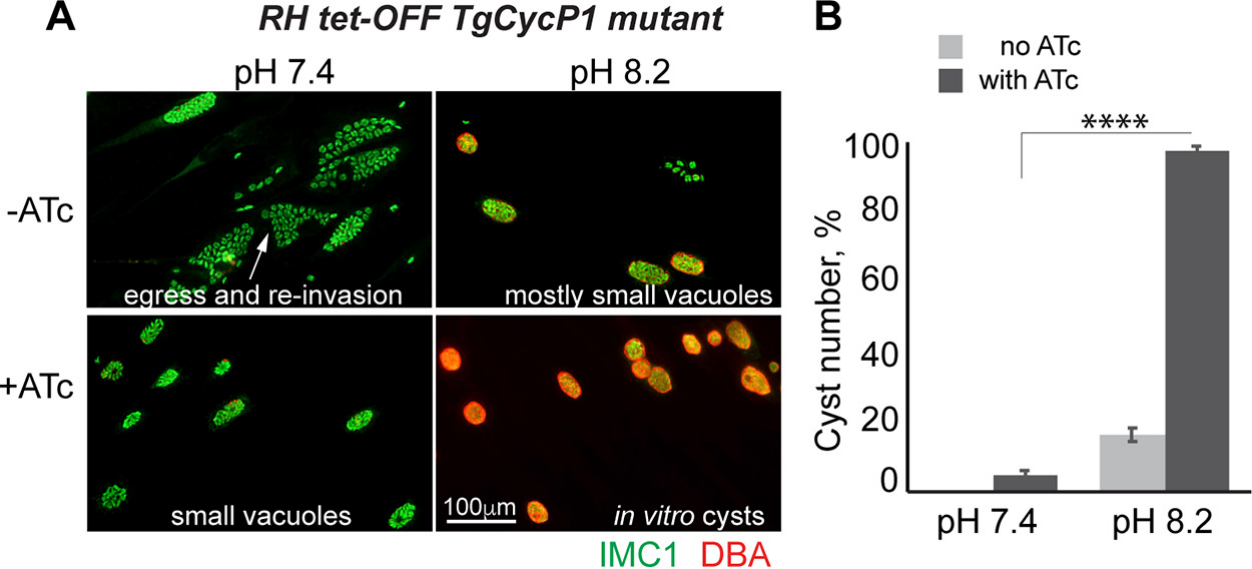
Expression of TgCycP1 negatively affects bradyzoite differentiation. (A) Immunofluorescent images of the RH Tet-OFF TgCycP1 mutant grown under tachyzoite (pH 7.4, 5% CO_2_, 2 days), bradyzoite differentiation (pH 8.2 ambient CO_2_, 3 days), and either in the presence or absence of 2 μM anhydrotetracycline (ATc). Individual parasites and tissue cysts were visualized with α-IMC1/α-rabbit IgG Alexa 488 and DBA/Texas Red Streptavidin 595, respectively. (B) Quantifications of cysts formed by RH Tet-OFF TgCycP1 mutant under indicated conditions. An unpaired *t* test returned a *P* value of 6.4 × 10^−8^.

We previously demonstrated that TgCycP1 functions as a G_1_ cyclin in the rapidly replicating RH tachyzoites (21). Using an RH TgCycP1 tet-OFF model we showed that TgCycP1 was required for entry into the S-phase, which corroborates the role of TgCycP1 in the regulation of the restriction checkpoint. Because bradyzoite maturation was accompanied by reduced replication rates and elongated G_1_ phase, we expected that TgCycP1 activity may not favor bradyzoite differentiation. To test this idea, we examined how depletion of the TgCycP1 cyclin affects the development of the poor cyst forming RH strain (Fig. 1A). Specifically, we tested the ability of RH TgCycP1 tet-OFF parasites to form *in vitro* cysts under factor-deficiency (induced by anhydrotetracycline [ATc]) and bradyzoite induction conditions (induced by alkaline pH 8.2). Tissue cysts were visualized using DBA (Dolichos biflorus agglutinin), which stains the cyst wall (13). In agreement with developmental incompetency, the stress-induced RH parasites did not efficiently form *in vitro* cysts (16.5%; Fig. 2A and B and Table S1; −ATc/pH 8.2) (29–31). Remarkably, ATc-dependent downregulation of TgCycP1 in the stress-induced RH parasites led to the nearly complete conversion of parasite vacuoles into DBA-positive *in vitro* cysts (97.5%; Fig. 2A and B and Table S1; +ATc/pH 8.2), indicating activation of the developmental program in TgCycP1-deficient RH parasites. These results demonstrated the negative effect of TgCycP1 expression on bradyzoite development and, together with its requirement for tachyzoite replication, confirmed the role of TgCycP1 in promoting cell division.

### TgCycP2 regulates G_1_ phase progression in ME49 tachyzoites.

**FIG 3:**
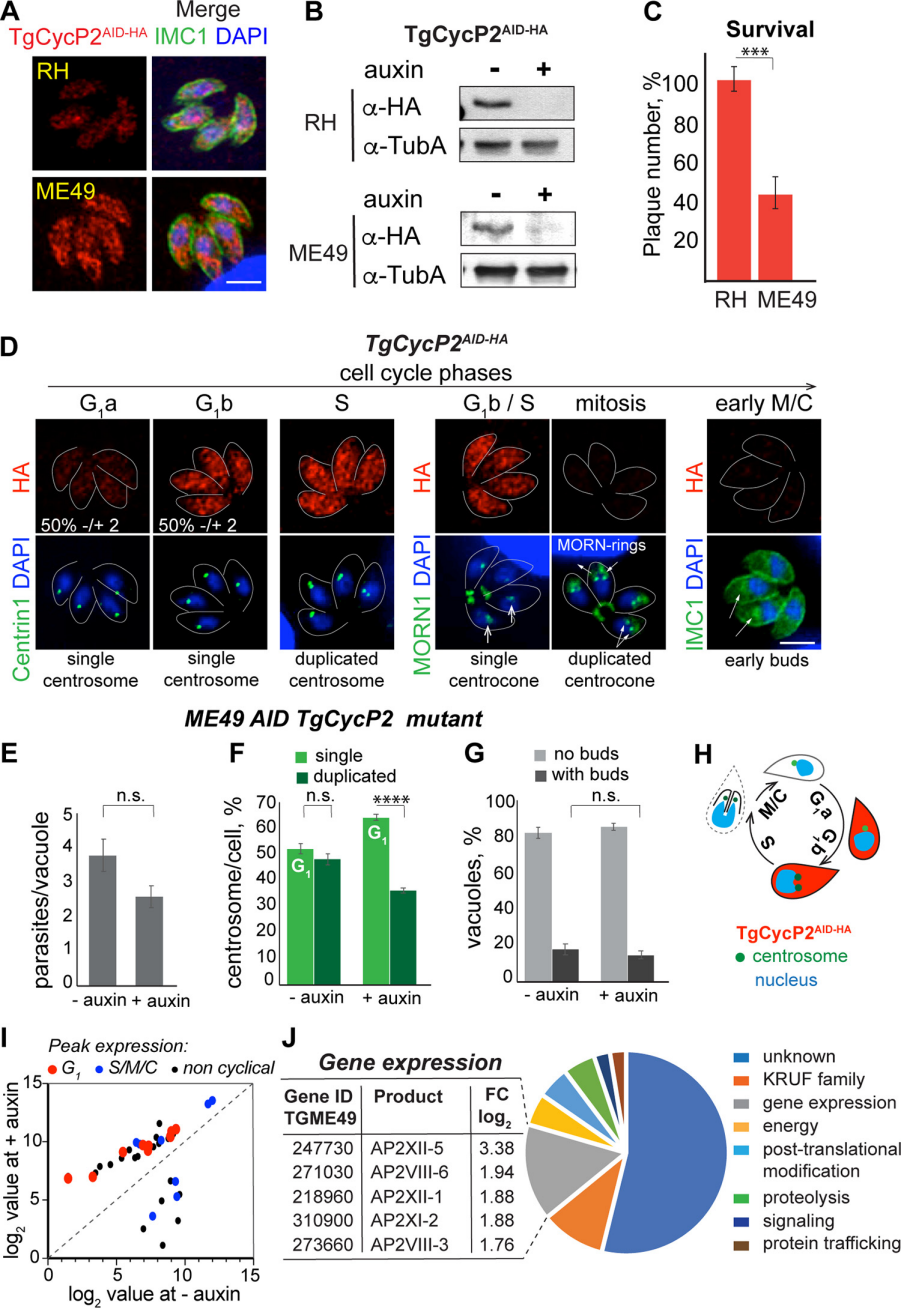
TgCycP2 regulates the G_1_ phase in type II ME49 parasites. (A) Immunofluorescent microscopy of TgCycP2^AID-HA^ expression in RHΔ*Ku80TIR1* and ME49Δ*Ku80TIR1* tachyzoites. TgCycP2^AID-HA^ was visualized with α-HA antibody (α-rat IgG Alexa Fluor 568) and costained with α-IMC1 (α-rabbit IgG Alexa Fluor488) and DAPI (nucleus). Scale bar 5 μm. (B) Immunoblot of the total lysates of RHΔ*Ku80TIR1* TgCycP2^AID-HA^ and ME49Δ*Ku80TIR1* TgCycP2^AID-HA^ parasites treated with vehicle (−) or 500 μM auxin (+) for 1 h. Western blots were probed with α-HA (α-rat IgG-HRP) to detect P-cyclins, and with α-Tubulin A (α-mouse IgG-HRP) to confirm equal sample loading. (C) Quantifications of plaques formed by RHΔ*Ku80TIR1* TgCycP2^AID-HA^ and ME49Δ*Ku80TIR1* TgCycP2^AID-HA^ lines. Parasites were grown with vehicle or 500 μM auxin for 6 (RH strain) or 9 (ME49 strain) days. The percentage of plaques formed by parasites in the presence of auxin relative to plaques formed by parasites treated with the vehicle is plotted on the graph. Data were analyzed by unpaired *t* test on three independent experiments (*P* = 4.7 × 10^−4^). (D) Immunofluorescence microscopy analysis of TgCycP2^AID-HA^ expression in ME49Δ*Ku80TIR1* tachyzoites. To reveal cell cycle-dependent expression, TgCycP2^AID-HA^ (α-HA/α-rat IgG Fluor 568) was costained with centrosomes (α-Centrin1/α-mouse IgG Fluor 488), centrocone (α-MORN1/α-rabbit IgG Flour 488) or alveolar protein IMC1 (α-IMC1/α-rabbit IgG Fluor 488). Cell cycle phases were determined based on the number and morphology of the reference structures indicated with arrows. Percentage of parasites with single centrosome, expressing or not expressing TgCycP2^AID-HA^ were calculated from 3 independent experiments. Mean value ± SD is shown. Scale bar 5 μm. (E) Degradation of TgCycP2 slows replication of ME49Δ*Ku80TIR1* tachyzoites. ME49Δ*Ku80TIR1* TgCycP2^AID-HA^ parasites were grown with vehicle or 500 μM auxin for 24 h and visualized with α-IMC/α-rabbit IgG Fluor 488 antibodies. The number of parasites per vacuole was quantified in three independent experiments. Mean values ± SD are plotted on the graph. The unpaired *t* test showed nonsignificant changes between the two conditions. (F) Quantification of IFA images of ME49Δ*Ku80TIR1* TgCycP2^AID-HA^ parasites treated with vehicle (−auxin) or with 500 μM auxin for 24 h and labeled with α-Centrin1 antibodies. The ratio of parasites with single (G_1_ phase, light green) or duplicated (S-phase/mitosis/budding, dark green) centrosomes is shown on the graph. The results of three independent experiments are shown. The unpaired *t* test showed nonsignificant changes at no auxin conditions and returned a *P* value of 4.2 × 10^−4^ in the presence of auxin. (G) Quantification of IFA images of ME49Δ*Ku80TIR1* TgCycP2^AID-HA^ parasites treated with vehicle (−auxin) or with 500 μM auxin for 24 h and labeled with α-IMC1 antibodies. The ratio of parasites with (light gray, see [E]) or without internal buds (dark gray) is plotted on the graph. An unpaired *t* test showed nonsignificant changes in the budding populations of parasites expressing or lacking TgCycP2. (H) Schematics of cell cycle-dependent TgCycP2 expression. Red cytoplasmic stain marks cell cycle phases of maximum TgCycP2 expression. Blue, nucleus; green dots, centrosomes. (I) Transcriptome analysis of ME49Δ*Ku80TIR1* TgCycP2^AID-HA^ tachyzoites. Transcripts with >1.5 log_2_ change in differential expression caused by auxin-induced TgCycP2^AID-HA^ degradation were sorted into G_1_, S/M/C, or noncyclical expression groups based on the predicted peak of the mRNA expression. A plot of the values at ± auxin conditions is shown. (J) The pie chart shows cellular pathways affected by TgCycP2 deficiency in ME49Δ*Ku80TIR1* tachyzoites. The table on the left lists the transcripts found in the gene expression category.

TgCrk2 forms a prominent complex with TgCycP2 in slower dividing ME49 but not in rapidly replicating RH tachyzoites (Fig. 1D), suggesting different functions in type I and type II parasites. To test this hypothesis, we built auxin-induced TgCycP2 degradation models in the developmentally incompetent RHΔ*Ku80TIR1* and competent ME49Δ*Ku80TIR1* strains (Fig. S1A to C) (32). Immunofluorescence microscopy and Western blot assays confirmed the expression of TgCycP2 in RH and ME49 tachyzoites, as well as the efficient downregulation of TgCycP2^AID-HA^ after an hour of treatment with 500 μM indole-3-acetic acid (auxin) (Fig. 3A and B). We also evaluated the requirement of TgCycP2 for the growth of RH and ME49 tachyzoites. Plaque assays revealed that the replication of ME49, but not RH tachyzoites, was dependent on TgCycP2 expression (Fig. 3C and Table S1). While all the auxin-treated TgCycP2^AID-HA^ RH parasites survived, degradation of TgCycP2^AID-HA^ in ME49 parasites resulted in a 60% reduction in plaque numbers.

Because TgCycP2 was required for proper replication of ME49 tachyzoites, we examined whether TgCycP2 affects cell cycle progression in ME49 tachyzoites. Immunofluorescence microscopy analysis showed that not all the asynchronously replicating clonal ME49 TgCycP2^AID-HA^ tachyzoites expressed TgCycP2^AID-HA^ (Fig. S2A). To find out if this variable TgCycP2 expression correlated with different cell cycle stages, we costained TgCycP2^AID-HA^ with T. gondii cell cycle markers. To identify parasites in the G_1_ phase, mitosis, and undergoing budding we costained and quantified TgCycP2-positive tachyzoites (α-HA) with centrosomes (α-Centrin1), centrocone/basal rings (α-MORN1), and surface alveoli (α-IMC1) (Fig. 3D) (33–35). Centrosome duplication marks the transition from G_1_ to the S-phase, which permits the identification of the G_1_ parasites (35, 36). We found that only 50% of tachyzoites in the G_1_ phase expressed TgCycP2^AID-HA^ (Fig. 3D and Table S1; single centrosome), suggesting that TgCycP2 emerges in the mid-G_1_ phase. The centrocone, an intranuclear compartment for the spindle, is formed in the late G_1_ stage, expanded in S-phase, and is duplicated in mitotic anaphase (22, 33, 35, 37). We established that TgCycP2 was downregulated in mitosis and budding stages because tachyzoites with a duplicated centrocone (α-MORN1) or with internal buds (α-IMC1) expressed significantly less TgCycP2^AID-HA^ (Fig. 3D and Fig. S2A). Thus, we concluded that TgCycP2 was a cyclical factor maximally expressed in the late G_1_ and S-phases of the tachyzoite cell cycle (Fig. 3H).

To determine the mechanism of the ME49 tachyzoite dependence on the TgCycP2 expression, we first measured replication rates of ME49 tachyzoites expressing (−auxin) or deficient (+auxin) in TgCycP2^AID-HA^. After 24 h, TgCycP2-depleted parasites divided slower than those expressing TgCycP2 (vacuole of 2 versus 4 parasites, respectively) (Fig. 3E and Table S1). To find out if TgCycP2 controls progression through the G_1_ phase in ME49 tachyzoites, we evaluated the changes in the duration of G_1_ caused by TgCycP2^AID-HA^ degradation (+auxin). Quantification of TgCycP2^AID-HA^ ME49 parasites with single (G_1_) and duplicated (S-phase, mitosis, and early budding) centrosomes revealed that TgCycP2-deficient parasites remain in the G_1_ phase longer than parasites expressing TgCycP2 (Fig. 3F and Table S1). At the same time, the lack of TgCycP2 did not affect the duration of the budding phase (Fig. 3G and Table S1). Thereby, we concluded that the longer division time of TgCycP2-deficient tachyzoites was due to a prolonged G_1_ phase.

To identify pathways regulated by TgCycP2, we performed a global transcriptome analysis of ME49 TgCycP2^AID-HA^ tachyzoites grown in the presence or absence of auxin (Table S4). RNA-seq analysis revealed upregulation of G_1_ transcripts in TgCycP2-deficient parasites (Fig. 3I) confirming the role of TgCycP2 in the G_1_ phase. Proteins with unknown functions represented a major category of the genes affected by the lack of TgCycP2 cyclin (Fig. 3J and Table S4). Downregulation of TgCycP2 significantly changed levels of the factors involved in the control of gene expression. We detected upregulation of five AP2 DNA-binding factors (Fig. 3J and Table S4). Three of them, AP2XI-2, AP2XII-1, and AP2XII-5, were components of a recently identified MORC complex that controls gene expression associated with parasite development (38). Altogether the cell cycle and the global transcriptome analyses showed that TgCycP2 regulates the G_1_ phase in developmentally competent ME49 tachyzoites. Our results revealed substantial differences in TgCycP2 function in RH and ME49 tachyzoites, which may explain the various replication rates and developmental capacity of the RH and ME49 parasites.

### Cyclin TgCycP2 was cyclically expressed in developing bradyzoites.

**FIG 4:**
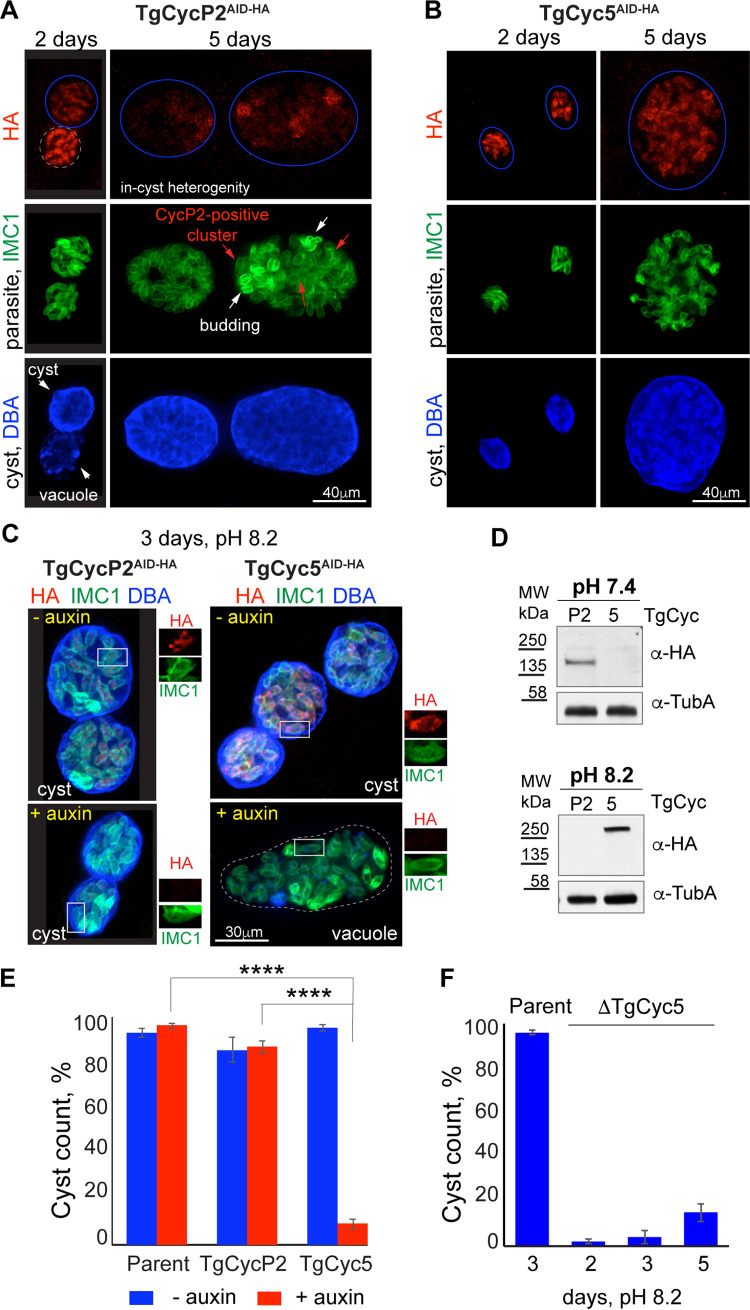
TgCycP2 and TgCyc5 play opposite roles in T. gondii differentiation. (A) IF analysis of ME49Δ*Ku80TIR1* TgCycP2^AID-HA^ mutant differentiation. Images show cyst expansion from day 2 to day 5 in a pH 8.2 medium. TgCycP2^AID-HA^ was detected with α-HA/α-rat IgG Fluor 568. Individual parasites were labeled with α-IMC1/α-mouse IgG Fluor 488 and the cyst wall was visualized using DBA/AMCA Streptavidin 350. Clusters of parasites undergoing the budding process are indicated with arrows on the IMC1 graph. (B) IFA images of day 2 and day 5 *in vitro* cysts formed by the ME49Δ*Ku80TIR1* TgCyc5^AID-HA^ mutant in a pH 8.2 medium. TgCyc5^AID-HA^ was labeled with α-HA/α-rat IgG Fluor 568, parasites with α-IMC1/α-mouse IgG Fluor 488, and cyst wall with DBA/AMCA Streptavidin 350. (C) IFA images of ME49Δ*Ku80TIR1* TgCycP2^AID-HA^ and ME49Δ*Ku80TIR1* TgCyc5^AID-HA^ mutants grown for 3 days in pH 8.2 medium with vehicle (−auxin) or with 500 μM auxin. The cyclins, parasites, and cysts were labeled with α-HA/α-rat IgG Fluor 568, α-IMC1/α-mouse IgG Fluor 488, and DBA/AMCA Streptavidin 350, respectively. The enlarged image on the right of each subfigure shows the expression or degradation of the corresponding AID-3xHA-fused cyclin. (D) Western blot of the total lysates of ME49Δ*Ku80TIR1* TgCycP2^AID-HA^ and ME49Δ*Ku80TIR1* TgCyc5^AID-HA^ tachyzoites (pH 7.4 medium for 2 days) and bradyzoites (at pH 8.2 medium for 3 days). Western blots were probed with α-HA (α-rat IgG-HRP) to detect cyclins, and with α-Tubulin A (α-mouse IgG-HRP) to confirm equal loading of lysates. (E) Quantification of cysts formed by the parental ME49Δ*Ku80TIR1* parasites and ME49Δ*Ku80TIR1* TgCycP2^AID-HA^ and ME49Δ*Ku80TIR1* TgCyc5^AID-HA^ mutants grown for 3 days in the pH 8.2 medium with vehicle (−auxin) or with 500 μM auxin. Cysts were identified by costaining of cyst wall (DBA/AMCA Streptavidin 350) and parasites (α-IMC1/α-mouse IgG Fluor 488). Bars show a mean value ± SD from three independent experiments. Unpaired *t* test returned *P* values of 3.6 × 10^−8^ and 8.9 × 10^−10^. (F) Quantification of cysts formed by the parent and ME49Δ*Ku80TIR1*Δ*TgCyc5^AID-HA^* mutant grown in pH 8.2 medium for the indicated time. Cysts were identified by costaining of the cyst wall (DBA/AMCA Streptavidin 350) and parasites (α-IMC1/α-mouse IgG Fluor 488). Bars show a mean value ± SD from three independent experiments. Note a steady temporal increase of cyst number formed by TgCyc5-deficient parasites.

To find out whether TgCycP2 plays a role in parasite development, we examined the ME49 TgCycP2^AID-HA^ strain under bradyzoite induction conditions. Parasites were monitored for 5 days in an alkaline medium where developing cysts displayed a highly heterogenous pattern of TgCycP2 expression similar to that observed in the tachyzoite stage (Fig. 4A). Costaining with antibodies against alveolar protein IMC1 (a marker of budding/cytokinesis) showed that budding parasites did not express cyclin TgCycP2 suggesting a similar cell cycle oscillation of TgCycP2 during bradyzoite development. Considering the extension of the G_1_ phase during development and the emergence of TgCycP2 in late G_1_ (Fig. 3H), we expected reduced levels of TgCycP2 in bradyzoites. Western blot of a 3-day bradyzoite population confirmed a significant decrease in TgCycP2^AID-HA^ expression (Fig. 4D). Judging by the number of cysts formed by ME49 parasites expressing (−auxin) or deficient in TgCycP2 (+auxin), the loss of TgCycP2 did not have a significant effect on bradyzoite development *in vitro* (Fig. 4E and Table S1). However, the TgCycP2-deficient cysts were smaller in size confirming that TgCycP2 contributes to bradyzoite replication (Fig. 4C, Fig. S2B, and Table S1).

### Cyclin TgCyc5 was required for bradyzoite differentiation *in vitro*.

The TgCyc5 was a novel atypical cyclin that formed a complex with the restriction checkpoint kinase TgCrk2 strictly under bradyzoite development conditions (Fig. 1D). To examine the function of TgCyc5, we generated RHΔ*Ku80TIR1* TgCyc5^AID-HA^ and ME49Δ*Ku80TIR1* TgCyc5^AID-HA^ strains for conditional TgCyc5 expression (Fig. S1B and C). We did not detect any measurable amount of TgCyc5 in type I or type II tachyzoites and the loss of TgCyc5 did not affect the tachyzoite growth (Fig. S2C and D and Table S1). Global gene expression analysis showed elevated levels of TgCyc5 mRNA under the high-pH stress conditions, suggesting TgCyc5 was involved in bradyzoite development (ToxoDB). To determine if upregulation of TgCyc5 mRNA results in elevated protein expression, we examined ME49 TgCyc5^AID-HA^ parasites under alkaline growth conditions. In agreement with mRNA upregulation, TgCyc5^AID-HA^ protein was detected on day 2 and was steadily expressed over 5 days of the *in vitro* cyst development (Fig. 4B and D). Further examination revealed that auxin-induced TgCyc5 deficiency had a dramatic effect on the dynamics of cyst formation. By 3 days of growth in an alkaline medium, more than 90% of vacuoles of TgCyc5-deficient parasites fail to convert into DBA-positive cysts (Fig. 4C and E and Table S1), inferring that TgCyc5 plays an essential role in bradyzoite development. Using a CRISPR approach we also generated a direct TgCyc5 knockout line of ME49 parasites (Fig. S2D to F). Similar to what was observed in the auxin-induced knockdown model, elimination of TgCyc5 did not affect the replication of ME49Δ*TgCyc5* tachyzoites but suppressed the *in vitro* cyst maturation (Fig. 4F and Table S1). Interestingly, the lack of TgCyc5 cyclin did not completely block cyst formation: around 10% of TgCyc5-negative vacuoles successfully converted into cysts after 3 days in pH 8.2 medium (Fig. 4E and F and Table S1). It suggested that removal of the bradyzoite cyclin TgCyc5 caused a severe delay rather than a failure of the cyst formation. Indeed, the time course of ME49Δ*TgCyc5* development revealed the number of cysts gradually increased over 5 days of differentiation (Fig. 4F and Table S1). Due to continued lytic cycles of TgCyc5 deficient parasites, we could not evaluate cyst formation beyond 5 days of growth in an alkaline medium.

**FIG 5:**
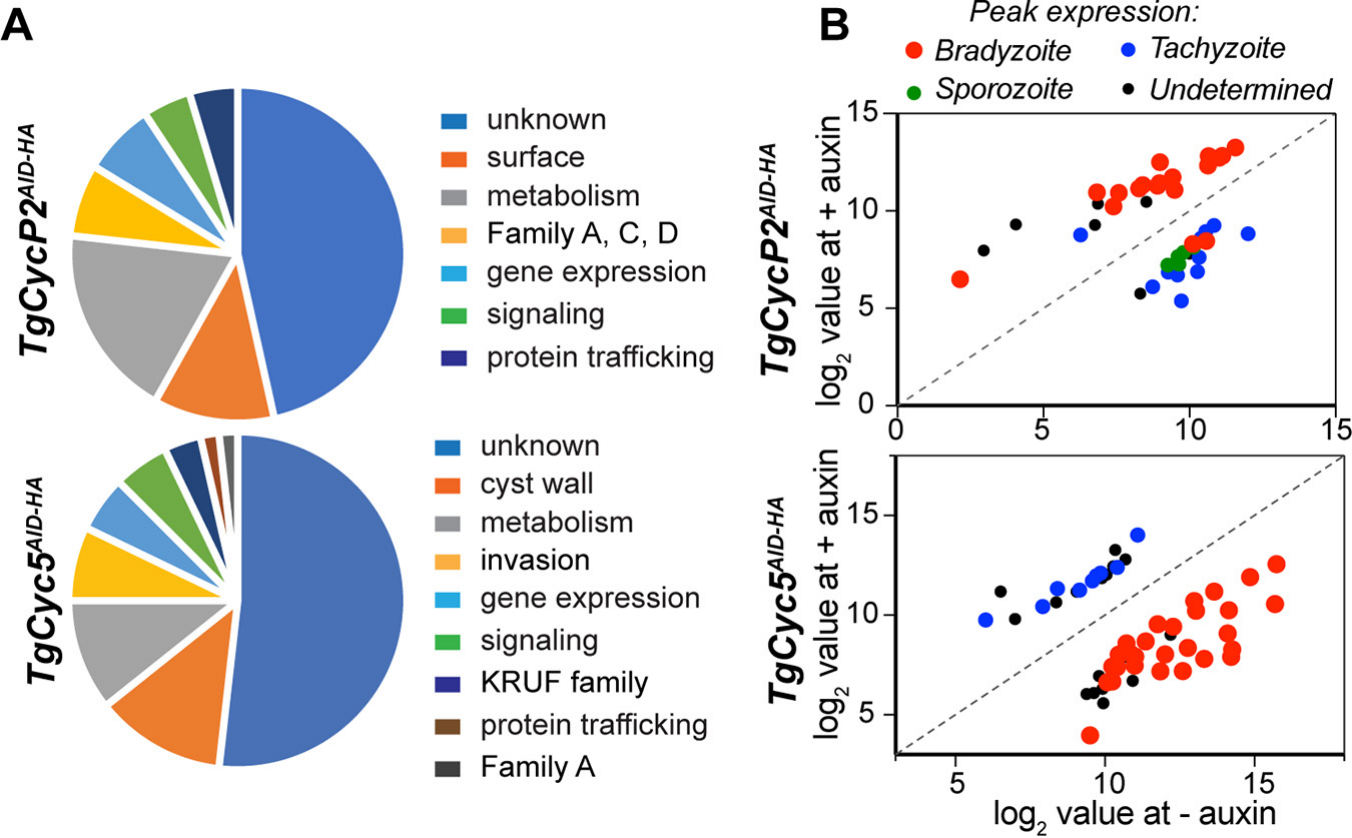
TgCyc5 is required for activation of the bradyzoite differentiation program. (A) The pie chart shows classes of transcripts differentially expressed upon conditional degradation of TgCycP2^AID-HA^ or TgCyc5^AID-HA^ cyclins during 3-day *in vitro* bradyzoite differentiation. (B) Transcripts with >1.5 log_2_ change expression in 3-day ME49Δ*Ku80TIR1* bradyzoites expressing (−auxin) or deficient (+auxin) in TgCycP2 or TgCyc5 are plotted on the graphs. Transcripts were sorted based on peak mRNA expression at different life stages (ToxoDB). The comparative analysis showed the opposite gene expression response to the loss of TgCycP2 and TgCyc5 cyclins.

We have established that TgCyc5 deficiency affects cyst wall maturation. To determine whether depletion of TgCyc5 also affected intracystic parasite development, we performed RNA-seq analysis of the auxin treated ME49 TgCyc5^AID-HA^ parasites after 3 days of growth in an alkaline medium (Table S4). Genome-wide changes in mRNA levels were compared to changes caused by the removal of TgCycP2 that did not perturb cyst wall formation (Fig. 4C). We found that most of the changes affected genes encoding proteins with unknown functions, and the second-largest category consisted of transcripts of metabolic and surface proteins, including cyst wall components (Fig. 5A and B). Analysis of stage-specific transcripts showed upregulation of canonical bradyzoite markers, such as Bag1, LDH2, and Eno1 in TgCycP2, and not the TgCyc5 transcriptome (Fig. 5B and Table S4). Although we detected a significant overlap between TgCycP2- and TgCyc5-dependent sets of genes (16.5% in common), the common genes showed opposite expression in TgCycP2 and TgCyc5 deficient parasites (Fig. 5B, red dots). Importantly, the pool of bradyzoite genes affected by TgCyc5 degradation was not limited to cyst wall components but also included metabolic and signaling factors, confirming that TgCyc5 was required for the physiological conversion of tachyzoites into bradyzoites (Table S4). Furthermore, we detected upregulation of tachyzoite-specific transcripts, which supported increased replication of TgCyc5 deficient parasites characteristic of the tachyzoite stage (Fig. 5B, blue dots). The functional assays together with global transcriptome analysis corroborated the developmental defect of TgCyc5 deficient ME49 parasites and confirmed the opposing roles for cyclins TgCycP2 and TgCyc5 in T. gondii development. Our results suggest that TgCycP2 promotes and TgCyc5 represses parasite replication. At the same time, TgCyc5 was critically needed for efficient bradyzoite differentiation.

### *Toxoplasma* restriction checkpoint controlled the parasite development in isolated rat brain cells.

**FIG 6:**
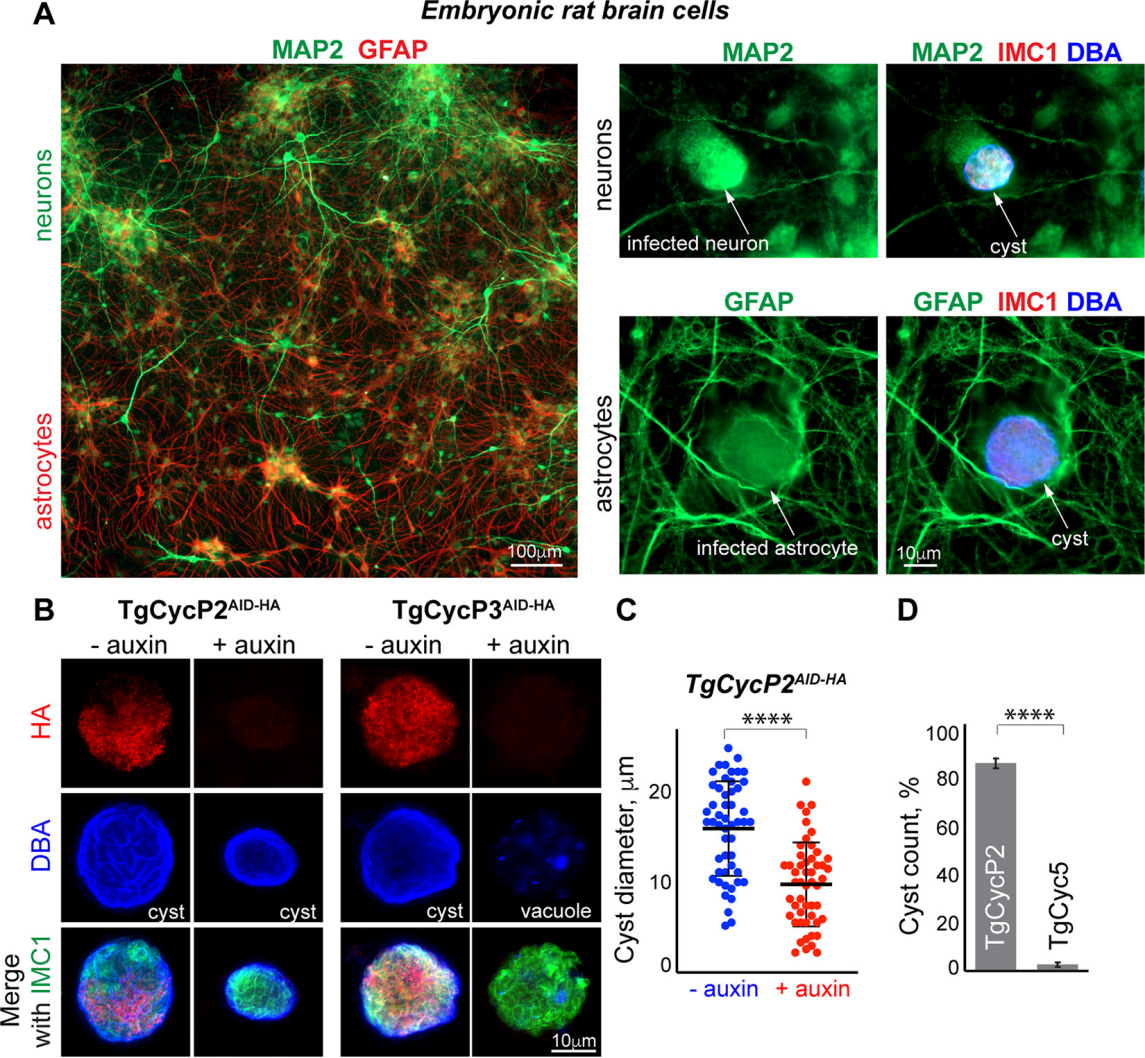
Function of the TgCycP2 and TgCyc5 in rat brain cells. (A) Costaining of the primary brain cells isolated from 18-day embryonic rats (E18). Neurons were detected using an antibody against microtubule-associated protein 2 (α-MAP2/α-chicken IgG Fluor 488). Copurified astrocytes were detected using an antibody targeting glial fibrillary acidic protein (α-GFAP/α-mouse IgG Fluor 568). Spontaneous cysts (DBA/AMCA Streptavidin 350) formed by ME49Δ*Ku80TIR1* parasites (α-IMC1/α-mouse IgG Fluor 568) in the isolated primary neurons (α-MAP2/α-chicken IgG Fluor 488) or astrocytes (α-GFAP/α-mouse IgG Fluor 488) are shown on the right. (B) Immunofluorescent images of the ME49Δ*Ku80TIR1* TgCycP2^AID-HA^ and ME49Δ*Ku80TIR1* TgCyc5^AID-HA^ cysts in embryonic rat neurons. The 3-day cysts/vacuoles formed in the presence or absence of 500 μM auxin are shown. *Toxoplasma* cyclins, individual parasites, and cysts were labeled with α-HA/α-rat IgG Fluor 568, α-IMC1/α-mouse IgG Fluor 488, and DBA/AMCA Streptavidin 350, respectively. (C) Quantification of the cyst sizes formed by ME49Δ*Ku80TIR1* TgCycP2^AID-HA^ mutant in rat neurons after 3 days of growth with vehicle or 500 μM auxin. Cyst diameter is plotted. Black lines indicate a mean value ± SD. The unpaired *t* test returned a *P* value of 3.6 × 10^−9^. (D) Quantification of the cysts formed by the ME49Δ*Ku80TIR1* TgCycP2^AID-HA^ and ME49Δ*Ku80TIR1* TgCyc5^AID-HA^ mutants after a 3-day infection of the rat brain cells in the presence of vehicle (−auxin) or 500 μM auxin. Cysts were identified by lectin binding with DBA/AMCA Streptavidin 350 and individual parasites with α-IMC1/α-mouse IgG Fluor 488. Cysts were quantified in 10 random microscopic fields. Bars show a mean value ± SD of the three independent experiments. An unpaired *t* test returned a *P* value of 8.3 × 10^−7^.

Previous *in vivo* studies and studies of the primary brain cultures identified neurons and astrocytes as the prevalent brain cells that support bradyzoite development (39–42). To determine whether the restriction checkpoint regulators control development in a system that more closely resembles *in vivo* infections, we examined TgCycP2^AID-HA^ and TgCyc5^AID-HA^ ME49 mutants in isolated brain cells. We adopted the published protocols and purified neurons and astrocytes from the brains of 18-day-old rat embryos (Fig. 6A, left) (43). Brain cell cultures were infected with ME49 parent, and cyst conversion was monitored for 3 days. We detected the spontaneous formation of DBA-positive cysts in MAP2-positive neurons and GFAP-positive astrocytes (Fig. 6A, right). To find out how the lack of individual cyclins affects spontaneous bradyzoite differentiation, infected brain cells were incubated in the presence or absence of auxin. Corroborating our findings in the alkaline model, TgCycP2 and TgCyc5 played distinctive roles in bradyzoite conversion in brain cells. TgCycP2 displayed a heterogenous pattern of intracystic expression similar to its expression in tachyzoites (Fig. 6B). The TgCycP2-negative cysts (+auxin) were smaller in size, confirming the cyclin’s role in bradyzoite replication (Fig. 6B and C and Table S1). Comparable to the high pH-stress model, TgCyc5 cyclin was upregulated in the infected brain cells, while the induced TgCycP5^AID-HA^ knockdown or TgCycP5 knockout severely obstructed differentiation of bradyzoites (Fig. 6B and D, Fig. S2F). The TgCyc5-deficient parasites formed fewer *in vitro* cysts than parasites expressing TgCyc5 (Fig. 6D and Table S1). Altogether, we have established opposite functions for TgCycP2 and TgCyc5 in distinct cell culture models of T. gondii development and, while promising, these findings will need to be confirmed in animal models of toxoplasmosis.

## DISCUSSION

The tight control of replication ensures the survival of T. gondii in the changing environment of different hosts. To complete its life cycle, a parasite must transmit from an intermediate to a definitive host, which requires substantial progeny generated by rapidly dividing tachyzoites. While the healthy immune system of the intermediate host eliminates most of the circulating tachyzoites, a small portion of tachyzoites gradually converts into the largely quiescent bradyzoites suitable for transmission into their definitive hosts, felids. The disbalance of division rates severely affects transmission rates and consequently, parasite survival. Reduced replication of tachyzoites lowers the chances to produce enough transmissible tissue cysts, while increased replication of bradyzoites results in cyst reactivation that threatens the host endurance. Despite its vital importance, little is known about the T. gondii growth-to-rest switch that controls stage transitions. In the current study, we discovered a molecular mechanism that links external signals to central cell cycle machinery, thereby determining whether the parasite divides (tachyzoite) or enters a quiescent state (bradyzoite).

The restriction checkpoint is a universal G_1_ mechanism that licenses eukaryotic cell division (44, 45). According to a recently suggested model, the R-point represents a window of opportunity whose duration is predetermined by external conditions (45, 46). Depending on the environment, the R-point either permits cell entry into the DNA replication phase (S-phase) that warrants cell division or retains cells in the G_1_ state that allows reprograming to fit into new growth conditions. Therefore, favorable conditions make the restriction checkpoint passage short and the cell division rapid, while unfavorable conditions extend the window of opportunity (longer G_1_ phase) slowing the rate of cell division. The concept of the restriction checkpoint echoes the mechanism of tachyzoite-to-bradyzoite conversion in T. gondii (11). Rapidly dividing tachyzoites grow at the most favorable conditions that promote a quick R-point passage, while developing bradyzoites divide progressively slower. Deeper in their development, bradyzoites spend more time within the window of opportunity critically needed to perform G_1_ functions, such as changes in epigenetic coding and gene expression (14, 19, 38, 47, 48). The completion of the cell cycle translates altered gene expression into a new metabolic state that enables bradyzoite survival.

**FIG 7:**
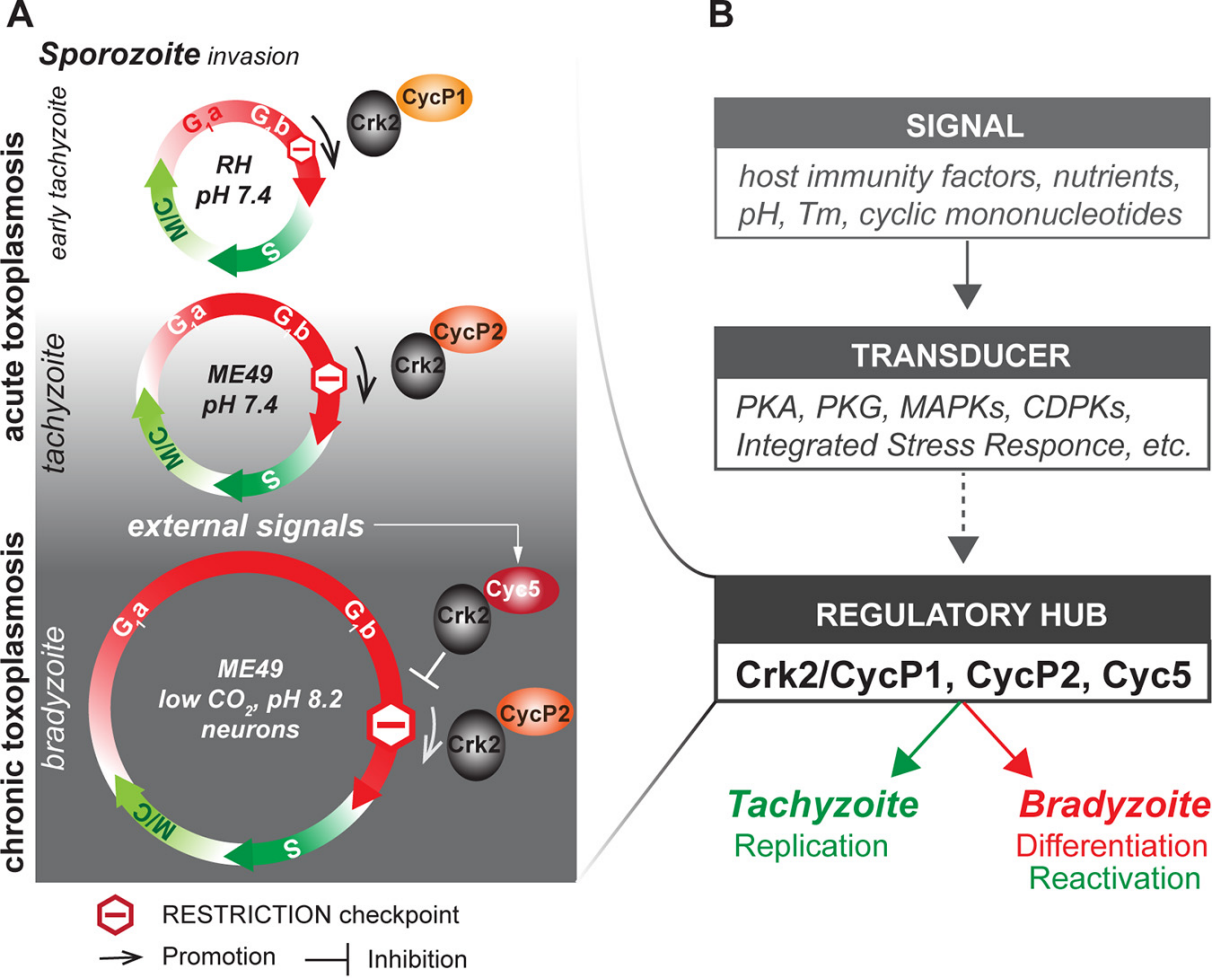
Model of restriction checkpoint regulation in T. gondii. (A) Schematics show the central cell cycle mechanisms that control T. gondii progression through intermediate life cycle stages. Cell cycles are drawn as color arrow circles: growth phase, G_1_a/G_1_b (red); DNA replication phase, S (dark green); mitosis coupled to cytokinesis (budding), M/C (light green). Note the relative extension of the G_1_ phase during bradyzoite development. Tachyzoite to bradyzoite conversion is regulated by alternative TgCrk2/Cyclin complexes operating at the restriction checkpoint (stop sign) in the G_1_ phase. The complex of the G_1_ kinase TgCrk2 with TgCycP1 promotes rapid cell division of early tachyzoites by quickly passing parasites through the R-point. In preparation for development, tachyzoites’ slow division was caused by the replacement of the highly active TgCrk2/TgCycP1 with moderately active TgCrk2/TgCycP2 complexes. Developmental (external) signals activate the expression of bradyzoite-specific TgCyc5, which in complex with TgCrk2, acts as a repressor of the R-point progression. T. gondii strains and *in vitro* conditions representing specific life cycle stages are indicated in the corresponding cell cycle diagrams. (B) Integration of the restriction checkpoint into the current model of the T. gondii development. Key factors that induce (signals), transduce (transducer), and activate (regulatory hub) tachyzoite-to-bradyzoite conversion are shown. Various signaling kinases and the integrated stress response (ISR) directly or indirectly activate/inhibit the restriction checkpoint function, which culminates in the execution of alternative differentiation programs. Dashed arrows mark putative connections.

Based on the published findings and our discovery of novel restriction checkpoint machinery in *Toxoplasma*, we propose a model that links disease dynamics to the core cell cycle mechanisms (Fig. 7A). Our model also explains the changing cell cycle architecture of bradyzoites toward gradual prolongation of the G_1_ phase (15). We believe that T. gondii actively employs the restriction checkpoint to control how soon parasites initiate DNA replication and complete the division cycle. Because modulation of Cdk activity is a major mechanism that acts in conventional checkpoints (45, 49), we propose that three novel T. gondii cyclins differentially control the R-point kinase TgCrk2, which is abundantly expressed in tachyzoites and bradyzoites. Our findings suggest that, in tachyzoites, cyclins TgCycP1 and TgCycP2 act as the TgCrk2 activators and promote the R-point passage. Given the dominant expression and absolute dependence of rapidly dividing RH tachyzoites on TgCycP1, we concluded that TgCycP1 functions as the most prominent TgCrk2 activator (Fig. 7, top). The TgCycP1 mRNA expression data across strains and conditions suggests that the laboratory-adapted RH strain likely mimics the early tachyzoite stage that emerges from sporozoites upon initial host infection (ToxoDB) (50, 51). Because the TgCrk2/TgCycP2 complex was detected only in the slower dividing ME49 parasites, we believe that TgCycP2 imposes restrictions on TgCrk2 activity leading to a longer G_1_ period (Fig. 7, middle). If needed, the elongated window of opportunity in type II tachyzoites can be used to change their developmental program. Corroborating this model, our global transcriptome analysis revealed the impact of TgCycP2 on the regulation of gene expression machinery in the ME49 tachyzoites (Fig. 3J). Developmental signals trigger upregulation of the repressive cyclin TgCyc5, resulting in bradyzoites that were retained at the R-point until a threshold of the TgCrk2 activity is achieved (Fig. 7, bottom). Passage through the restriction checkpoint takes a progressively longer time during development that manifests as a quiescence of the mature bradyzoite. Bradyzoite differentiation is a Coccidian-specific process, which explains the lack of the TgCycP2 and TgCyc5 orthologs in other apicomplexan parasites (22). Thus, it is tempting to suggest that this unique R-point module of T. gondii based on the alternative regulation of a single Cdk kinase by multiple cyclins in various capacities had evolved to accommodate a graded bradyzoite development.

Previous studies identified several signaling pathways associated with stage conversion in T. gondii (52). It has been demonstrated how various extracellular signals, including host immune factors, are transduced either directly or via second messengers to various signaling kinases (5, 31, 53–57). The critical role of the integrated stress response (ISR) and unfolded protein response (UPR) pathways in bradyzoite maturation has also been demonstrated (48, 52, 58). These stress response systems control preferential mRNA maturation and protein production, and in conjunction, were predicted to control the expression of the recently identified regulators of parasite differentiation, such as BDF1 and stage-specific AP2 factors (59–61). How is the restriction checkpoint module integrated into the multifactorial network of *Toxoplasma* differentiation? Because the R-point controls the uniform outcome of development, namely, the reduction of replication rates, the T. gondii R-point most likely functions as a regulatory hub (Fig. 7B). The restriction checkpoint machinery receives signals from a variety of signaling pathways and uniformly responds by changing the rates of replication. Although further studies are needed to find out how external signals are delivered to the R-point machinery, we can speculate a few scenarios. For example, signaling kinases PKA and PKG may modulate the activity of the constitutively expressed kinase TgCrk2 by direct phosphorylation, while the ISR system may control the signal-induced expression of the R-point cyclins. Stress-activated IRS may repress translation of the tachyzoite cyclin TgCycP1 and, concurrently, favor the expression of the bradyzoite cyclin TgCyc5. Our study identified the core machinery of the tachyzoite-to-bradyzoite switch. However, the variety of incoming signals and the nuances of response associated with different hosts and environments, suggest that the R-point network is much wider. In the future, addressing the molecular composition of the restriction checkpoint as well as an examination of its functional links with known signaling pathways will help us to find more efficient ways to control toxoplasmosis, particularly in the drug-resistant chronic stage.

## MATERIALS AND METHODS

### Parasite cell culture.

Parasites were grown in human foreskin fibroblasts (HFF) as described (62). Transgenic and mutant parasite lines are derivatives of the RHΔ*Ku80*Δ*hxgprt AtTIR1* and ME49Δ*Ku80*Δ*hxgprt AtTIR1* parasite strains (32). Host and parasite strains were tested mycoplasma free (MP Biomedicals). All parasite lines created in the study are listed in Table S5.

### Rat embryonic brain cell cultures and infections.

The primary rat brain cells were purified from 18-day embryos (E18) of Wistar rats (Rattus norvegicus) of both sexes as previously described (43). Isolated cells were plated on the poly-d-Lysine (Gibco) coated coverslips to a density of 5 × 10^4^ cells per 1.9cm^2^ (43). Brain cells were cultured in the neurobasal medium (Invitrogen) supplemented with B-27 and 2 mM Glutamax (Gibco) and the medium was changed every 7 days. Brain cell cultures were examined by immunofluorescence microscopy using cell-type-specific antibodies. Staining with α-MAP2 (ThermoFisher) and α-Gfap (Agilent) revealed enrichment of the isolated cultures with neurons and astrocytes, respectively. Seven-day cultures were used in all the experiments. Typical infection was initiated with T. gondii ME49 transgenic tachyzoites at 1:5 MOI (1 × 10^5^ parasites per 1.9 cm^2^ well). Parasite growth and development were monitored for 3 days and evaluated by immunofluorescence microscopy. All animal studies described herein were reviewed and approved by the ethics committee at our institution.

### Phylogenetic analysis.

Protein sequences were obtained from eupathDB and NCBI databases and shown in Table S3. The analysis involved 32 amino acid sequences of the P-type cyclins from apicomplexans Toxoplasma gondii, Hammondia hammondia, Plasmodium falciparum, Babesia bovis, Cryptosporidium parvum, alveolate Vitrella brassicaformis, Chromera velia, and selected representatives of P-cyclins from fungi (Saccharomyces cerevisiae, Aspergillus nidulans, and Candida viswanathii) and plant Arabidopsis thaliana. We generated multiple sequence alignment using the MUSCLE algorithm in R package ‘msa’ version 1.4.3 (63). The Bayesian information criterion (BIC) was used to search for the best amino acid substitution models. The substitution model ‘JTT’ was selected to initially build an unrooted tree using neighbor-joining (64). Then, we optimized the length of the generated tree by maximum likelihood (65). The 100 times bootstrap was performed to validate the optimized edges. We used R package ‘phangorn’ version 2.8.0 for phylogeny and ‘ggtree’ version 3.2.0 to draw the final phylogenetic tree (66, 67).

### Construction of the transgenic strains.

Primers and transgenic strains created in the current study are listed in Table S5.

### RHΔ*Ku80*Δ*hxgprt AtTIR1* TgCycP2^AID-HA^ and RHΔ*Ku80*Δ*hxgprt AtTIR1* TgCyc5^AID-HA^ strains.

First, we built the pLIC-mAID-3xHA-Hxgprt vector. An AvrII/NdeI fragment containing a 3xHA epitope of pLIC-3xHA-Hxgprt plasmid was replaced with a complementary AID-3xHA fragment from the pTUB1-YFP-mAID-3xHA-DHFR-TS-Hxgprt2 vector (32). DNA fragments encompassing the 3′-end of the gene of interest were then amplified by PCR and cloned into the pLIC-mAID-3xHA-Hxgprt vector digested with PacI endonuclease by the Gibson assembly method. The resulting pLIC-TgCycP2-mAID-3xHA-Hxgprt and pLIC-TgCyc5-mAID-3xHA-Hxgprt constructs were linearized and transfected into the RHΔ*Ku80*Δ*hxgprt AtTIR1* parent (32).

### ME49Δ*Ku80*Δ*hxgprt AtTIR1* TgCycP2^AID-HA^ and ME49Δ*Ku80*Δ*hxgprt AtTIR1* TgCyc5^AID-HA^ strains.

To introduce mAID-3xHA-epitope to the C terminus of TgCycP2 and TgCyc5 proteins in the ME49Δ*Ku80*Δ*hxgprt AtTIR1* parent, we used CRISPR/Cas9 approach as previously described (32). To introduce a double DNA break in the 3’UTR of the tagging gene locus, we created gene-specific gsRNA CRISPR/Cas9 plasmids by site-specific mutagenesis of pSAG1:CAS9-GFP U6:gsUPRT plasmid generously provided by L. David Sibley (68). Tagging cassette containing the selection marker *hxgprt* flanked with 40 nt gene-specific sequences was amplified by PCR. A P-cyclin-specific tagging cassette and a sgRNA CRISPR/Cas9 plasmid were cotransfected into the ME49Δ*Ku80*Δ*hxgprt AtTIR1* parent.

### ME49ΔKu80ΔAtTIR1ΔTgCyc5 strain.

The TgCyc5 knockout cassette was built in the pLIC-3xHA-Hxgprt plasmid. A 1,058nt of 5’UTR and 968 nt of TgCyc5 3’UTR were PCR amplified and cloned sequentially upstream (into HindIII site) and downstream (into SacI site) of the *hxgprt* cassette using the Gibson assembly approach. The resulting *3’UTR_TgCyc5/hxgprt/5’UTR_TgCyc5* knockout cassette and two TgCyc5-specific gsRNA CRISPR/Cas9 plasmids were electroporated into the ME49Δ*Ku80*Δ*hxgprt AtTIR1* parent.

### Parasite transfection.

10 million freshly lysing RH tachyzoites or 20 million ME49 tachyzoites were mixed with up to 30 μg DNA in 100 μL of the cytomix buffer and electroporated with Amicon electroporator (Lonza). Transfected parasites were allowed to recover in fresh HFF monolayers for 24 h before drug selection. To obtain a clonal population the drug-resistant polyclonal populations were cloned by limited dilution. No less than three individual clones were confirmed by PCR and sequencing for proper recombination at the locus and protein expression by Western blot and immunofluorescent microscopy.

### Western blotting.

Filter-purified parasites were washed in PBS and collected by centrifugation. Total lysates were obtained by resuspending the parasite pellets in the Leammli loading dye, heated at 95°C for 10 min, and briefly sonicated. After separation on the SDS-PAGE gels, proteins were transferred onto nitrocellulose membrane and probed with monoclonal α-HA (rat 3F10, Roche Applied Sciences), α-myc (mouse, Cell Signaling Technology), and α-Tubulin A (mouse 12G10, kindly provided by Jacek Gaertig, University of Georgia) antibodies. After incubation with secondary HRP-conjugated anti-mouse or anti-rat antibodies, proteins were visualized by enhanced chemiluminescence detection (PerkinElmer).

### Immunofluorescence analysis.

Confluent HFF cultures on glass coverslips were infected with parasites for indicated times and under specified growth conditions. Infected monolayers were fixed, permeabilized, and incubated with antibodies as previously described (69). The following primary antibodies were used; rat monoclonal α-HA (clone 3F10, Roche Applied Sciences), rabbit α-Myc (clone 71D10, Cell Signaling Technology), mouse α-Centrin (clone 20H5, Millipore Sigma), rabbit polyclonal α-MORN1 (centrocone and basal complex stains, kindly provided by Marc-Jan Gubbels, Boston College, MA) and rabbit and mouse polyclonal α-IMC1 (parasite shape and internal daughter bud stains, kindly provided by Gary Ward, University of Vermont, VT). All Alexa-conjugated secondary antibodies (ThermoFisher) were used at a dilution of 1:500. To visualize the *in vitro* cysts, we used biotinylated Dolichos biflorus agglutinin (DBA, Vector Laboratories) and the corresponding streptavidin-conjugated fluorophores. Chicken polyclonal antibodies against neuronal microtubule-associated protein 2 (MAP2) were used to identify isolated rat neurons (PhosphoSolutions). Coverslips were mounted with Aquamount (ThermoFisher), dried overnight at 4°C, and viewed on Zeiss Axiovert Microscope equipped with 100× objective. Images were collected and processed first using Zeiss Zen software, then in Adobe Photoshop 2021 using linear adjustment when needed.

### Plaque assay.

Confluent HFF monolayers in a six-well dish format were infected with 100 (RHΔ*Ku80*Δ*hxgprt AtTIR1* strain derivatives) or 300 (ME49Δ*Ku80*Δ*hxgprt AtTIR1* strain derivatives) freshly lysing parasites. If needed, cultures were treated with either a vehicle (EtOH) or 500 μM IAA (auxin) to trigger an AID-protein degradation. Plaques developed after 6 days (RH strain derivatives) or 9 days (ME49 strain derivatives) were processed for indirect immunofluorescence microscopy using rabbit polyclonal α-*Toxoplasma* antibody (Abcam) and counted. At least three biological replicates of each assay were performed.

### *In vitro* bradyzoite assay.

The HFF monolayers were infected with freshly lysed tachyzoites at 1:2 MOI for 2 h. To induce bradyzoite differentiation, the normal growth medium was replaced with a bradyzoite induction medium (DMEM without bicarbonate supplemented with 25 mM HEPES, 5% fetal bovine serum, pH 8.2). Infected HFF monolayers were incubated at 37°C in the ambient air chamber for the indicated time. Tachyzoite to bradyzoite conversion was evaluated by conversion of the tachyzoite vacuoles into the DBA-positive *in vitro* cysts.

### RNA-seq analysis sample preparation.

Total RNA was extracted from the purified tachyzoites and bradyzoites of the ME49Δ*Ku80*Δ*hxgprt AtTIR1* strain and derivatives with TRIzol according to the manufacturer’s instructions (Invitrogen). The RNA integrity was verified using Agilent High Sensitivity D1000 ScreenTape in an Agilent 2200 TapeStation System (Agilent Technologies). Two biological replicates were analyzed.

### RNA-Seq library preparation and sequencing.

For each sample, one microgram of total RNA was treated with 1 U of DNase I (Application Grade, Invitrogen) to remove the residual genomic DNA, and the DNase-treated RNA was used as an input for the construction of the RNA-seq library using IlluminaTruSeq Stranded mRNA Library Preparation kit (Illumina) according to the manufacturer’s instructions. The mRNA was purified from total RNA using oligonucleotide (dT)-conjugated magnetic beads. Purified mRNAs were fragmented at 94°C for 8 min and first-strand cDNA was synthesized using random hexamer primers and SuperScriptII Reverse Transcriptase (Invitrogen) in First-Strand Synthesis Act D Mix (Illumina). Second strand cDNA synthesis was subsequently performed in Second Strand Marking Master Mix (Illumina). After adenylation of the 3′ ends, RNA Adapter Index codes were ligated to their respective cDNA samples. To select ~280bp cDNA fragments, the libraries were purified with the AMPure XP system (Beckman Coulter). To enrich adaptor-ligated cDNA fragments, the libraries were PCR-amplified (PCR Master Mix and PCR Primer Cocktail, Illumina) and purified with the AMPure XP system. The quality of the libraries was confirmed using Agilent High Sensitivity D1000 ScreenTape in an Agilent 2200 TapeStation System (Agilent Technologies). The cDNA concentration was measured by qPCR and with a Qubit DNA assay kit in a Qubit 2.0 Fluorometer (Thermo Scientific). A total of 12 cDNA libraries were applied to NextSeq 500/550 Mid Output kit v2.5 (150 cycles) and sequencing on Illumina NextSeq System to obtain 5 million of 75 bp paired-end sequencing reads per sample. RNA sequencing was performed at the University of South Florida (USF) Genomics Program Sequencing Core.

### Data analysis.

Transcriptomes were aligned using Hisat version 2.1.0 using the ToxoDB version 41 as a reference genome (70). The gene expression values were quantified with Stringtie and transcripts per million (TPM) were used for downstream analyses (71). Differential expression analysis was conducted with DESeq 3.1.2 using R 3.6.1 (72). For each cyclin, we fit a model with three factors: strain, growth conditions (pH), and factor expression (± auxin). We conducted a log-ratio test to see whether the removal of the treatment variable had a significant effect on the model fit. We used a cutoff of 0.1 for the adjusted *P* value for consideration of differentially expressed genes.

### Proteomic analysis sample preparation.

Each sample was prepared from no less than 2 × 10^9^ parasites. Tachyzoites or developing bradyzoites were collected by filtration and centrifugation after growth under indicated conditions. Total proteins were extracted by incubation in the phosphate-buffered saline solution with 263 mM NaCl, 0.25% NP-40, and a proteinase-phosphatase inhibitors cocktail (Thermo Fisher). TgCrk2^HA^ or TgCrk2^myc^ complexes were isolated on α-HA or α-Myc magnetic beads (MblBio). The efficiency of immunoisolation was verified by Western blot of TgCrk2HA or TgCrk2myc kinases.

### Mass spectrometry.

Protein samples were processed for mass spectrometry-based proteomic analysis using filter-aided sample preparation (FASP). Briefly, proteins are reduced using dithiothreitol (DTT), alkylated with iodoacetamide (IAA), buffer exchanged with urea followed by ammonium bicarbonate, and finally digested with Trypsin/Lys-C overnight at 37°C. Peptides were eluted and desalted using C18 SPE columns (Waters) with a vacuum manifold and dried in a vacuum concentrator. Peptides were separated using a 75 μm x 50 cm C18reversed-phase-UHPLC column (Thermo Scientific) on an Ultimate 3000 UHPLC (Thermo Scientific) with a 120-minute gradient (2 to 32% acetonitrile with 0.1% formic acid). Full MS survey scans were acquired at 70,000 resolution. Data-dependent acquisition (DDA) selected the top 10 most abundant ions for MS/MS analysis. Raw data files were processed in MaxQuant (www.maxquant.org) and searched against the current Uniprot Toxoplasma gondii ME49 protein sequence database. Search parameters include constant modification of cysteine by carbamidomethylation and the variable modification, methionine oxidation. Proteins were identified using the filtering criteria of 1% protein and peptide false discovery rate.

### Data analysis.

The sum of peptides counts is calculated for each protein. The cum up count for each assay is divided by the corresponding protein length (ToxoDB). The result value for each protein is normalized by dividing the value of the specifically isolated TgCrk2 kinase. The normalized value from three assays is merged and trained in a Gaussian mixture model. The purpose of using the mixture model is to identify the potential or hidden component of the observed normalization peptide counts and to select the component where the corresponding protein exhibits higher peptide counts. The Gaussian mixture model was performed on log_2_ transformed normalized counts, and the posterior probability was calculated to determine the likelihood of a protein to be bound to TgCrk2. Proteins with a posterior probability higher than 0.5 and raw peptide counts of at least five were considered in complex with TgCrk2.

### Data accessibility.

The mass spectrometry proteomics data have been deposited to the ProteomeXchange Consortium via the PRIDE partner repository (http://proteomecentral.proteomexchange.org/cgi/GetDataset) with the data set identifier PXD033109 (73). The RNA-seq data were deposited at NCBI GEO (GSE200962).
