# Supplementary material for: Restriction Checkpoint Controls Bradyzoite Development in Toxoplasma gondii
Source: Microbiol Spectr. 2022 Jun 2;10(3):e00702-22. doi: 10.1128/spectrum.00702-22 (PMC9241953; doi:10.1128/spectrum.00702-22)
Supplement: SUPPLEMENTAL FILE 1 — Supplemental material. Download spectrum.00702-22-s0001.pdf, PDF file, 2.6 MB [file spectrum.00702-22-s0001.pdf]

Supplemental Data

Figure S1. Generation of auxin-induced degradation models for TgCycP2 and TgCyc5 cyclins in *T. gondii* RH and ME49 strains.

(A) Diagram shows the mechanism of auxin-induced degradation of AID-tagged proteins. In the absence of plant hormone auxin (IAA – indole-3-acetic acid) a Cyclin$^{mAID\text{-}3xHA}$ expression reaches endogenous levels. Addition of 500μM auxin (yellow star) promotes interaction of AID-modified Cyclins with F-box protein TIR1 (dark blue) and ubiquitin ligase SCF (light blue) resulting in rapid degradation of the modified Cyclin by the proteasome.

(B) Schematics for constructing AID-modified Cyclin genes. In the RH*ΔKu80TIR1* parent: Targeting plasmid included 3' fragment of the target genomic locus for the tagging Cyclin fused with mini-version of AID (AID), 3xHA (HA) epitopes and the drug-selection marker *hxgprt* gene (grey box). Recombination at the target locus is induced by the plasmid linearization with a unique endonuclease. In the ME49*ΔKu80TIR1* parent: Targeting cassette was produced by PCR and contains an AID, 3xHA-epitope and the *hxgprt* gene. To ensure specific knock-in, the PCR fragment carries 40bp ends specific for the 3' end of each *Cyclin* gene. A CRISPR double-strand break (DBS, green arrow) is achieved by co-transfection of a gene-specific gRNA-CAS9 plasmid [75]. Schematics also indicate relative position of primers used to confirm the proper Cyclin's knock-in event (panel C).

(C) DNA electrophoresis analysis of parental and transgenic strains. To confirm a proper modification of the *TgCycP2* and *TgCyc5* loci, the 3'end of the corresponding open reading frame was amplified using the indicated primer combination. Note that the 1+2 primer combination used to examine RH transgenic lines did not produce a PCR product due to large size of the integrated fragment (targeting plasmid, 7kb).

(D) Schematics for constructing the ME49*ΔKu80 AtTIR1ΔTgCyc5* strain. Targeting cassette containing *hxgprt* gene flanked with 1058 nt 3'UTR and 968 nt 5'URT of the TgCyc5 gene was produced by PCR. Two CRISPR double-strand breaks (DBS, green arrow) were introduced by co-transfection of two gene-specific gRNA-CAS9 plasmids [75]. Schematics also indicate relative position of primers used to confirm replacement of the *TgCyc5* locus with the *hxgprt* cassette (panel E).

(E) DNA electrophoresis analysis of the parent and ME49*ΔKu80 AtTIR1ΔTgCyc5* strain.

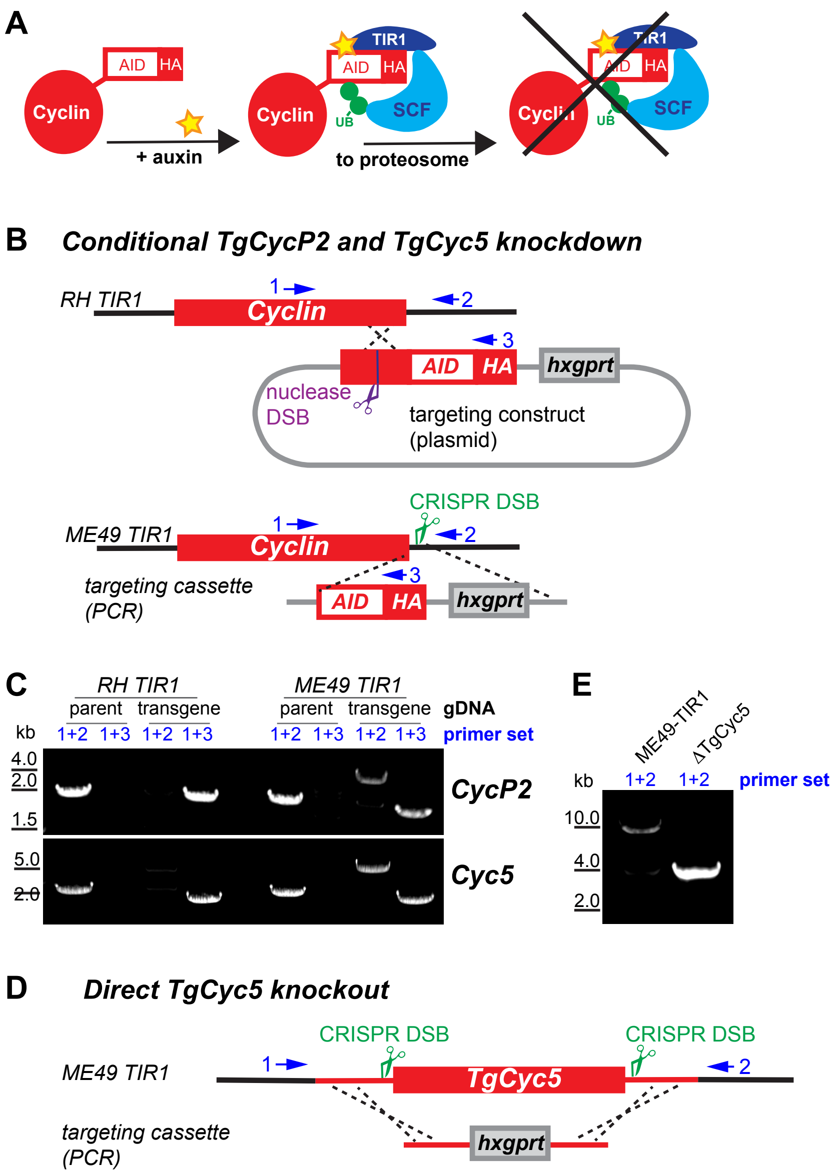
A
AID
HA
Cyclin
+ auxin
TIR1
SCF
UB
to proteosome
B
Conditional TgCycP2 and TgCyc5 knockdown
RH TIR1
Cyclin
AID
HA
hxgprt
nuclease
DSB
targeting construct
(plasmid)
CRISPR DSB
ME49 TIR1
targeting cassette
(PCR)
C
RH TIR1
ME49 TIR1
parent
transgene
gDNA
kb
primer set
1+2
1+3
4.0
2.0
1.5
5.0
CycP2
Cyc5
E
ME49-TIR1
ΔTgCyc5
10.0
D
Direct TgCyc5 knockout
TgCyc5

Figure S2. Role of TgCycP2 and TgCyc5 in tachyzoites and developing bradyzoites.

(A) IF microscopy analysis of TgCycP2^{AID-HA} expression in RH*ΔKu80TIR1* tachyzoites. Image depicts two vacuoles of parasites (grey dotted line) undergoing alternative cell cycle phases identified based on cell morphology and co-staining with cell cycle markers. To identify budding parasites, TgCycP2^{AID-HA} (α-HA/α-rat IgG Fluor 568) was co-stained with alveolar protein IMC1 (α-IMC1/α-rabbit IgG Fluor 488). Parasites in G_1/S phases have a single round nucleus (DAPI stain).

(B) Quantification of cyst size formed by ME49*ΔKu80TIR1* TgCycP2^{AID-HA} mutant after 3 days growth in bradyzoite induction medium (pH 8.2) with vehicle (- auxin) or 500 μM auxin (+ auxin). Cyst diameters are plotted. Black lines indicate a mean value -/+ SD. Unpaired t-test returned a p-value 6.3×10^{-4}.

(C) Immunofluorescent microscopy of TgCyc5^{AID-HA} expression in RH*ΔKu80TIR1* and ME49*ΔKu80TIR1* tachyzoites. TgCyc5^{AID-HA} (α-HA, α-rat IgG Alexa Fluor 568) was co-stained with α-IMC1 (α-rabbit IgG Alexa Fluor488) and DAPI. Scale bar 5μm.

(D) Quantifications of plaques formed by RH*ΔKu80TIR1* TgCyc5^{AID-HA} and ME49*ΔKu80TIR1* TgCyc5^{AID-HA} tachyzoites. Parasites were grown with vehicle or 500μM auxin for 6 (RH strain) or 9 (ME49 strain) days. Percentage of plaques formed by parasites in the presence of auxin relative to parasites treated with vehicle in three independent experiments is plotted on the graph.

(E) Western Blot analysis of the total lysates of ME49*ΔKu80TIR1* TgCyc5^{AID-HA} tachyzoites (pH 7.4 medium for 2 days) and bradyzoites (at pH 8.2 medium for 3 days), grown with (+) or without (-) 500 μM auxin. Western blots were probed with α-HA (α-rat IgG-HRP) to detect TgCyc5^{AID-HA}, and with α-Tubulin A (α-mouse IgG-HRP) to confirm equal loading of lysates.

(F) Immunofluorescent images of ME49*ΔKu80TIR1* TgCyc5^{AID-HA} parasites after 3 days growth in embryonic rat brain cells in the presence or absence of 500 μM auxin. Individual parasites and cysts were labeled with α-IMC1/α-mouse IgG Fluor 488 and DBA/Texas red Streptavidin 595, respectively.

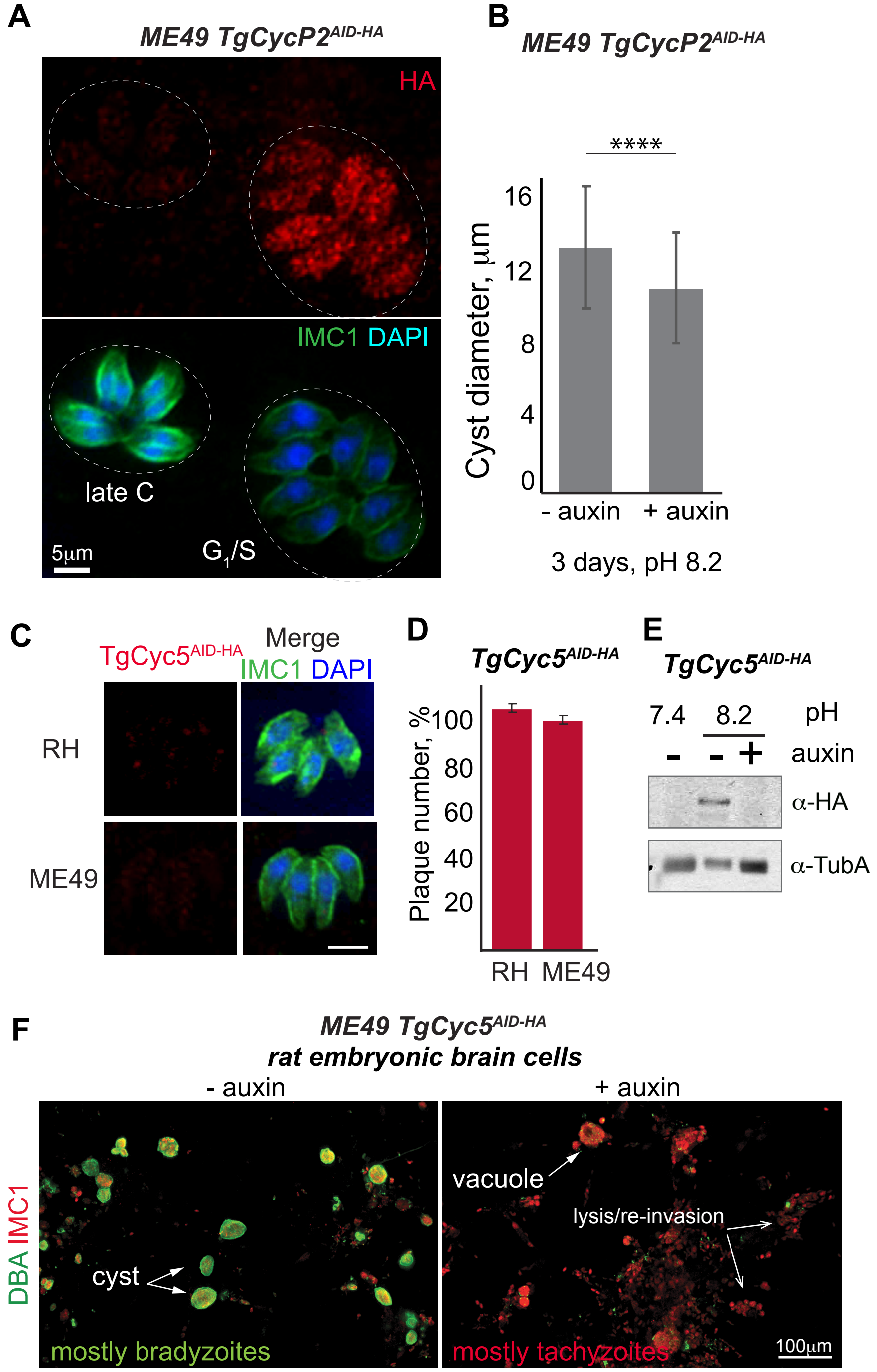
A
ME49 TgCycP2AID-HA
HA
IMC1 DAPI
late C
G1/S
5µm
B
ME49 TgCycP2AID-HA
****
Cyst diameter, µm
16
12
8
4
0
- auxin
+ auxin
3 days, pH 8.2
C
TgCyc5AID-HA
Merge
IMC1 DAPI
RH
ME49
D
TgCyc5AID-HA
Plaque number, %
100
80
60
40
20
RH
ME49
E
TgCyc5AID-HA
7.4
8.2
pH
-
-
+
auxin
α-HA
α-TubA
F
ME49 TgCyc5AID-HA
rat embryonic brain cells
- auxin
+ auxin
DBA IMC1
cyst
mostly bradyzoites
vacuole
lysis/re-invasion
mostly tachyzoites
100µm
